# Beyond αβ T cells: unlocking the potential of diverse immune cells in CAR modification

**DOI:** 10.1042/CS20256571

**Published:** 2026-01-21

**Authors:** Yangyang Gao, Feifei Guo, Min Li, Naifei Chen, Chao Niu, Jiuwei Cui

**Affiliations:** 1 Cancer Center, The First Hospital of Jilin University, Changchun, 130021, China

**Keywords:** chimeric antigen receptor, gamma delta T cells, immunotherapy, macrophages, NK cells

## Abstract

Chimeric antigen receptor (CAR) T cell therapy has emerged as a groundbreaking advancement in cancer immunotherapy, demonstrating remarkable success in treating hematologic malignancies. However, its application in solid tumors remains challenging. The complex manufacturing process and severe treatmentrelated toxicities further limit its broader clinical application. To address these challenges, researchers are investigating alternative CAR-engineered immune cells, including CAR-NK cells, CAR-γδ T cells, and CARmacrophages (CAR-M), which offer distinct advantages over conventional CAR-T therapy. Notably, CAR-NK and CAR-γδ T cells exhibit HLA-independent cytotoxicity, making them promising ‘off-the-shelf’ therapeutic options. Meanwhile, CAR-M not only phagocytose tumor cells and present antigens but also remodel the immunosuppressive tumor microenvironment. Despite their potential, these innovative therapies still face several challenges in clinical application. This review systematically summarizes recent advances in CAR-T cells, CAR-NK cells, CAR-γδ T cells, and CAR-M for cancer treatment, providing a comprehensive analysis of their respective strengths, limitations, and future optimization strategies to support the clinical translation of next-generation CAR-based immunotherapies.

## Introduction

Chimeric antigen receptor (CAR)-T cell therapy has transformed the landscape of cancer immunotherapy, achieving outstanding efficacy in the treatment of hematologic malignancies [1]. Although it can induce long-term remissions in refractory cases, its efficacy in solid tumors remains limited due to physical barriers, the immunosuppressive tumor microenvironment (TME), and tumor heterogeneity [2]. Additional limitations include complex manufacturing processes, high costs [3], and treatment-related adverse events mainly cytokine release syndrome (CRS) and neurological toxicity [4]. The immune system harbors diverse cell types with unique capabilities that may overcome limitations of conventional CAR-T therapy. Recent advances have enabled genetic engineering of alternative immune effectors including natural killer (NK) cells, macrophages, and γδ T cells, each offering distinct therapeutic advantages. CAR-NK cells demonstrate HLA-independent cytotoxicity, permitting allogeneic use without donor matching while maintaining solid tumor infiltration capacity with reduced toxicity risks [5]. γδ T cells uniquely bridge innate and adaptive immunity, exhibiting potent antitumor activity even in immunosuppressive microenvironments [6]. Macrophages provide multifunctional potential through simultaneous tumor phagocytosis, antigen presentation, and immune modulation [7]. These engineered alternatives not only address CAR-T manufacturing challenges but may offer more precise tumor targeting. However, successful clinical translation requires a deeper understanding of each cell type’s unique biology. This review summarizes current progress with CAR-engineered immune cells, examining their distinct antitumor mechanisms, recent genetic engineering breakthroughs, and persisting clinical challenges. By evaluating these developments, the review aims to clarify how alternative cellular therapies might surpass current CAR-T limitations and advance cancer treatment paradigms.

## The limitations of CAR-T cell therapy

CAR-T cell therapy, first developed by Kuwana et al. in 1987, has evolved significantly from its original conceptual framework [8]. The canonical CAR structure consists of three functional domains: an extracellular single-chain variable fragment (scFv) for antigen recognition, a transmembrane anchoring domain, and an intracellular signaling domain that combines CD3ζ activation with co-stimulatory signals from CD28 or 4–1BB [9]. This innovative design has translated into clinical success, with nine approved therapies globally, including six FDA-approved products [10-15] and three authorized in China [16-18], demonstrating particular efficacy in hematological malignancies [19,20].

**Figure 1:**
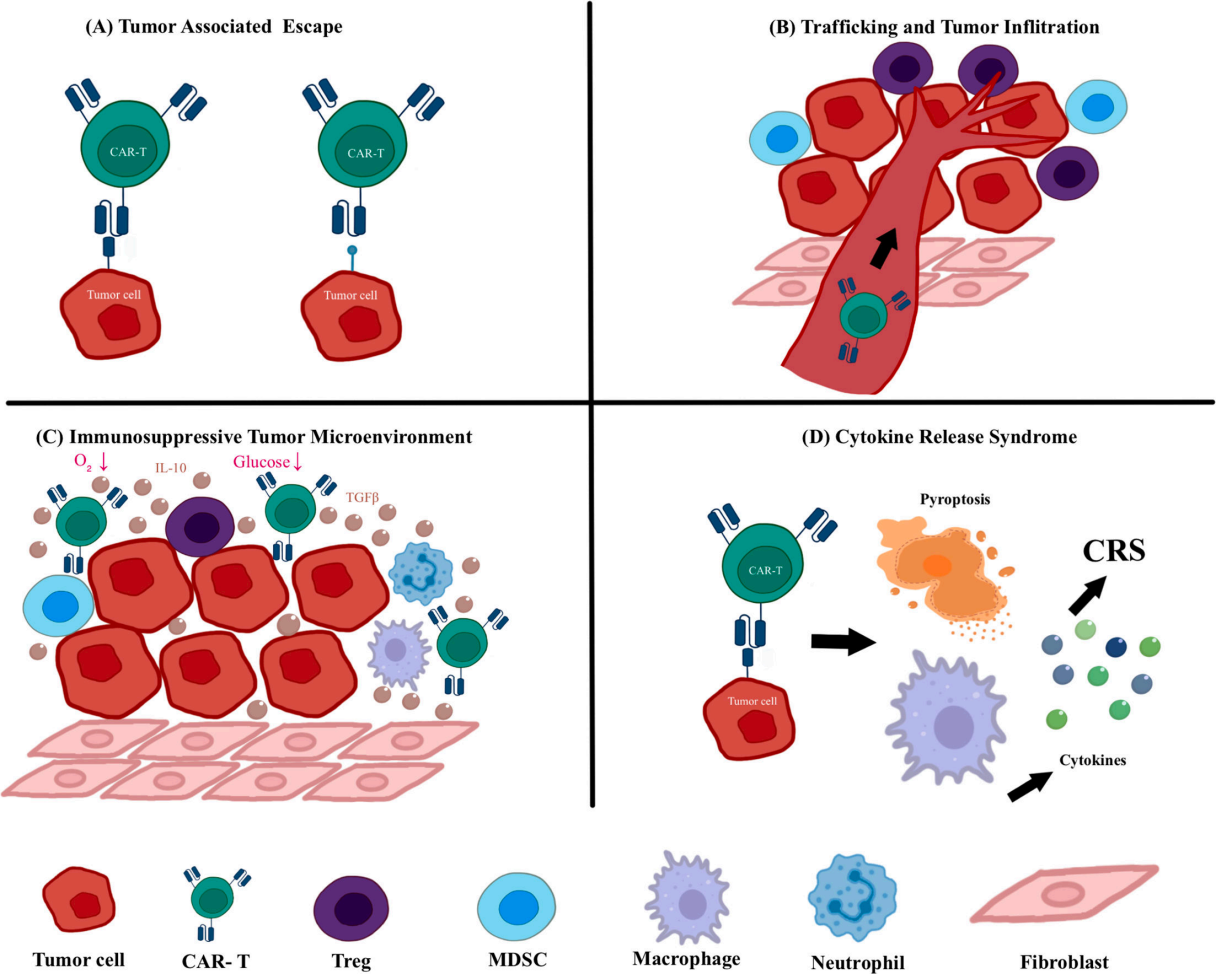
Current challenges in CAR-T cell therapy. (**A**) Tumor-associated antigen escape reduces CAR-T cell efficacy by allowing tumor cells to evade recognition. (**B**) Poor trafficking and infiltration into solid tumors limit CAR-T cell accumulation at tumor sites. (**C**) The immunosuppressive tumor microenvironment inhibits CAR-T cell activation and function through various soluble factors and suppressive cell types. (**D**) Cytokine release syndrome (CRS) is a life-threatening systemic inflammatory response triggered by excessive immune activation.

Despite these advances, several critical challenges hinder broader application. The autologous nature of current CAR-T therapies results in substantial costs and prolonged manufacturing timelines due to the required T cell activation, viral transduction, and expansion processes [21,22]. Furthermore, treatment-related toxicities, particularly CRS, pose significant clinical risks through potential multi-organ dysfunction [23,24]. The efficacy in solid tumors remains limited due to multiple factors including antigen escape from heterogeneous expression patterns [25,26], poor tumor infiltration caused by dense fibrotic stroma [27], and a suppressive TME characterized by regulatory T cells, myeloid-derived suppressor cells, and metabolic constraints such as hypoxia and nutrient depletion that collectively promote T-cell exhaustion [28,29]. These biological barriers underscore the need for innovative approaches to enhance tumor penetration, overcome immunosuppression, and improve metabolic adaptation (Figure 1). Future development of CAR-based therapies should focus on engineering next-generation immune effectors capable of addressing these limitations to expand therapeutic potential in a broader spectrum of cancers. Recent advances in CAR-engineered immune cell therapy have expanded beyond conventional αβ T cells to include promising alternatives like CAR-NK cells, CAR-γδ T cells, and CAR-M, each offering unique advantages for cancer treatment.

## Distinctive CAR design across immune cell types

The structural configurations and genetic modification strategies for CAR-engineered immune cells exhibit significant variations depending on the cellular platform.

CAR-NK constructs retain the core CAR-T framework but add NK-specific features to broaden antigen recognition. Ongoing optimization of signaling domains further improves persistence and cytotoxicity. For CAR-γδ T cells, evolving designs have progressed from initial CD3ζ-based architectures to innovative nonsignaling CAR (NSCAR) formats that maintain tumoricidal activity while minimizing exhaustion. CAR-M development has focused on phagocytic functionality, integrating macrophage-specific signaling pathways that confer unique microenvironmental advantages.

### CAR design for NK cells

CAR-NK cells utilize a receptor structure fundamentally similar to CAR-T cells but incorporate strategic modifications to enhance NK-specific functionality.

These cells target tumor-associated antigens including CD19, HER2, and BCMA. They also leverage innate receptors like NKG2D, which recognize multiple stress-induced ligands (MICA/B, RAET1/ULBP) on tumor cells [30]. This dual recognition capability has been successfully translated into clinical applications, with NKG2D-based CAR constructs demonstrating efficacy in both lung cancer preclinical models [31] and clinical trials for metastatic colorectal cancer [32]. The transmembrane domain composition significantly influences cytotoxic activity, as evidenced by studies showing NKG2D-derived domains confer superior target cell lysis compared with other NK receptor variants like NKp46 or CD16 [33]. Intracellularly, CAR-NK cells initially used T-cell signaling elements (CD3ζ, 4–1BB). Recent designs add NK-specific scaffolds like DAP10/DAP12 and co-stimulatory molecules DNAM-1 and 2B4 to improve persistence and cytotoxicity [34]. Particularly noteworthy is the inclusion of 2B4, a SLAM family member that significantly potentiates cytokine secretion and degranulation responses [35]. Further engineering innovations have yielded chimeric receptors combining NKG2D and CD244 signaling pathways, demonstrating promising results in castration-resistant prostate cancer models [36], along with strategies enabling autonomous cytokine secretion to improve tumor homing and microenvironmental survival [37-39].

Despite these technological advances, the field still faces important challenges including an incomplete understanding of NK-specific signaling requirements and a lack of clinical validation for many combinatorial approaches. Advancing next-generation CAR-NK therapies require sustained attention to receptor engineering strategies that align with the intrinsic biology of NK cells, while preserving the superior safety characteristics that set them apart from CAR-T cells.

### CAR design for γδ T cells

The structural evolution of CAR-γδ T cells has progressed through several generations to optimize their antitumor efficacy and safety profile. First-generation CAR-γδ T cells used CD3ζ signaling domains. Rischer et al. showed that CD19- and GD2-targeted constructs lysed tumors effectively and up-regulated markers like CD69 and IFN-γ [40]. Subsequent second-generation improvements incorporated co-stimulatory molecules like CD28, with Deniger et al. demonstrating enhanced persistence and antitumor activity both *in vitro* and *in vivo* [41]. To address off-target toxicity concerns, innovative chimeric co-stimulatory receptor (CCR) designs emerged, substituting traditional CD3ζ with DAP10 signaling domains. These GD2-DAP10 CAR-γδ T cells maintained potent tumor lysis while minimizing nonspecific cytotoxicity [42]. Recently, the unique signaling characteristics of γδ T cells have been widely investigated. Anderson et al. used single-cell mass cytometry to analyze CAR signaling networks and found that excessive CD3ζ signaling could lead to γδ T cell dysfunction. To mitigate this, they developed a signaling optimization strategy by replacing the signaling domain with gentler alternatives such as DAP10, enhancing antitumor activity while reducing side effects [43]. Most remarkably, NSCAR designs represent a paradigm shift by eliminating intracellular signaling entirely. These NSCAR-γδ T cells mediate tumor killing through antigen down-regulation while avoiding exhaustion—a particularly valuable feature for treating T cell malignancies [44]. This progression from first-generation to sophisticated NSCAR designs illustrates how structural innovations continue to expand the therapeutic potential of CAR-γδ T cells.

### CAR Design for macrophages

The design of CAR-M builds upon fundamental CAR-T principles while incorporating macrophage-specific modifications. Although the CD3ζ intracellular domain can initiate phagocytic signaling through ITAM-mediated Syk kinase recruitment, similar to early CAR-T experience, this single signaling domain proves insufficient for optimal macrophage activation. To enhance functionality, researchers have developed specialized phagocytic CARs (CAR-Ps) featuring cytoplasmic domains from Megf10 and FcRɣ, which significantly improve phagocytic activity [45]. Simultaneously, Niu et al. designed CAR-M targeting chemokine receptor 7 (CCR7) to eliminate a newly identified immunosuppressive cell population characterized by high lipid droplet content and elevated CCR7 expression (LD^hi^ CCR7^hi^). Among the constructs tested, the CAR-M containing the intracellular signaling portion of MerTK demonstrated the greatest tumoricidal activity [46]. In another approach, researchers utilized CAR-M with a CD147 activation domain, which, upon recognizing the tumor antigen HER2, activates the receptor to trigger CD147’s intracellular signaling and increase matrix metalloproteinase (MMP) levels. Although CAR-147 macrophage therapy showed no effect *in vitro*, the infusion of CAR-147 macrophages significantly inhibited the growth of HER2-4T1 tumors in BALB/c mice. This effect could be attributed to CAR-147 macrophages reducing tumor collagen deposition, thereby promoting T-cell infiltration into the tumor tissue [47]. These advances highlight how macrophage-specific CAR engineering requires both adaptation of conventional CAR concepts and development of novel, myeloid-optimized signaling architectures to fully exploit their therapeutic potential.

**Figure 2:**
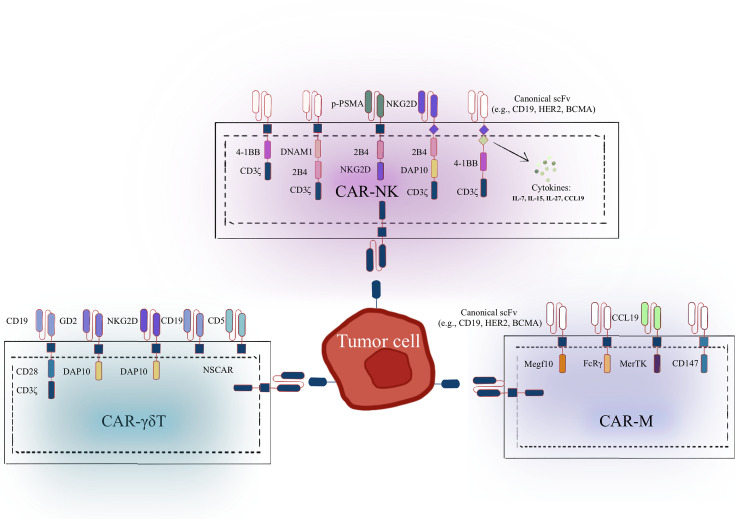
Structural diversity of CAR among NK, γδ T, and macrophage cells. Representative CAR designs that have been functionally validated in NK cells, γδ T cells, and macrophages. DAP10, DNAX activation protein 10; DNAM1, DNAX accessory molecule-1; Megf10, multiple EGF-like domains 10; MerTK, Mer tyrosine kinase; NKG2D, natural killer group 2D; NSCAR, nonsignaling CAR; p-PSMA, prostate specific membrane antigen-targeted polypeptide.

The structural design of CARs varies significantly across immune cell types, reflecting the distinct biological features and functional needs of NK cells, γδ T cells, and macrophages. CAR-NK constructs adapt the traditional CAR-T framework with NK-specific receptors and signaling domains to enhance cytotoxicity and broaden target recognition. CAR-γδ T designs have evolved from conventional signaling CARs to nonsignaling variants that preserve antitumor function while reducing exhaustion and off-target effects. In contrast, CAR-M integrate macrophage-centric signaling elements to drive phagocytosis and reshape the TME. These cell-specific innovations underscore the importance of tailored CAR designs to fully harness the therapeutic potential of each immune effector type (Figure 2).

### Limitations and future directions

Although CAR designs for NK cells, γδ T cells, and macrophages have made initial progress, most designs are directly adapted from CAR-T platforms without specific optimization for the unique biology of each cell type. Regarding CAR-NK cells, conventional T-cell signaling domains, including CD28 and 4–1BB, may not adequately support NK-specific activation and *in vivo* persistence, while the clinical validation of NK-specific signaling molecules such as DAP10, DAP12, or 2B4 remains limited. In CAR-γδ T cells, the integration between CAR signaling and endogenous γδ TCR responses is not well understood, and the potential for functional exhaustion under chronic antigen exposure remains unclear. CAR-macrophage designs are largely at the proof-of-concept stage, with insufficient data on how intracellular CAR domains regulate macrophage polarization, phagocytosis, and long-term survival. Future studies should prioritize cell type-specific CAR architectures, systematically compare signaling backbones, and validate functional consequences in clinically relevant tumor models to harness cell-specific advantages while addressing inherent limitations.

## Distinctive transduction approaches across immune cell types

Transduction methodologies similarly reflect cell-type specific requirements. While viral vectors remain predominant for CAR-NK and CAR-γδ T cells, their sensitivity to viral components has spurred development of alternative approaches including mRNA electroporation (EP) and lipid nanoparticles (LNPs) delivery. CAR-M presents particular transduction challenges due to macrophage resistance, addressed through specialized viral vectors like Ad5f35 and novel nonviral techniques. These cell-specific design philosophies and delivery strategies will guide future optimizations targeting enhanced structural precision, transduction efficiency, and therapeutic potency.

### Transduction approaches of CAR-NK

The introduction of CAR genes into immune cells typically relies on viral vectors with exogenous promoters. While studies in primary T cells demonstrate superior CAR expression with the EF1α promoter compared with CMV, UbiC, and PGK promoters [48], NK cells show distinct preferences—the SFFV promoter achieves optimal lentiviral titers and transduction efficiency in these innate lymphocytes [49]. To enhance NK cell engineering, researchers have developed structural vector modifications, including an innovative lentiviral vector (pCDH-CMV-MCS-P2A-copGFP-T2A-Puro) where replacement of the EF1 promoter with a P2A self-cleaving peptide sequence significantly boosted both CAR expression levels and tumoricidal activity in human NK cells [50]. These findings highlight the importance of cell type-specific optimization in CAR delivery systems, particularly when translating approaches between adaptive and innate immune effectors.

Three primary nonviral approaches have shown promise: EP, LNPs, and trogocytosis-mediated membrane transfer. EP represents a cost-effective method for transient CAR expression, demonstrating the ability to deliver both CAR and CCR7 genes into primary NK cells and NK-92 cell lines while significantly enhancing their cytotoxicity and migratory capacity [51]. Compared with DNA transfection, mRNA transfection is a more efficient and faster method for generating CAR-NK cells. Carlsten and colleagues successfully transfected NK cells using the mRNA EP method, inducing rapid and repeatable transgene expression in CAR-NK cells without negatively affecting cytotoxic function, viability, or phenotype [52]. LNPs are lipid vesicles with a homogeneous lipid core, constructed from four lipid components: ionizable lipids, cholesterol, auxiliary lipids, and polyethylene glycol (PEG) lipids [53]. One study encapsulated BCMA-CAR mRNA and CD19-CAR mRNA in LNPs, achieving CAR expression rates of 95% and 78% in NK cells, respectively [54]. Further research also demonstrated that nanoparticles can effectively introduce small interfering RNA into NK cells [55]. CAR-NK cells generated through the mRNA-LNP platform exhibit significantly enhanced cytotoxicity against CD19^+^ target cells [56]. Trogocytosis is an active process whereby donor and recipient cells transfer small membrane fragments via stable intercellular interactions [57]. Cho et al. demonstrated that K562 cell lines expressing high levels of anti-CD19 CAR could transfer these CARs into NK cells via trogocytosis [58]. Furthermore, some researchers have found that NK cell trogocytosis could impair the effectiveness of NK cell-based immunotherapy strategies. Li et al. showed that CAR activation promotes the transfer of CAR homologous antigens from tumor cells to NK cells [59]. This results in antibody self-recognition and antigen-mediated CAR reactivation on the NK cell surface, leading to fratricide and exhaustion. Modifying the CAR signaling domains may provide a potential solution to this issue, as studies have shown that trogocytosis has different effects on CARs with CD28 or 4–1BB signaling domains [59,60]. Potential strategies to regulate phagocytosis include drug targeting, dual CAR systems, and signaling domain modifications [61].

CRISPR/Cas9 technology has revolutionized cellular engineering by enabling precise genome editing through targeted DNA cleavage. This powerful tool has been successfully integrated with CAR transduction to enhance NK cell therapies. For instance, researchers have combined transposon engineering with CRISPR/Cas9 modification to develop CLL-1-targeting CAR-NK cells, which demonstrate superior tumor-killing efficiency compared with conventional approaches [62]. Beyond CAR integration, CRISPR-Cas9 offers potential for modifying exhausted NK cells within the challenging TME [63]. A deeper understanding of various patterns of senescence, exhaustion, and dysfunction observed in the TME—such as reduced effector cytokine production, impaired cytotoxicity, the presence of inhibitory cytokines, and receptor signaling dysregulation—may help in designing strategies to enhance NK cell function [64]. These applications position CRISPR-Cas9 as a transformative technology for designing next-generation NK cell immunotherapies with enhanced functionality and persistence.

### Transduction approaches of CAR-γδ T

The method used to introduce the CAR gene plays a critical role on the functionality and stability of CAR-γδ T cells. Common transfection strategies include viral vector transduction, EP, transposon systems, and CRISPR/Cas9 gene editing. Retroviral and lentiviral vectors are the most commonly used gene transduction tools, enabling efficient and stable gene integration. Capsomidis et al. used retroviral transduction to introduce GD2-CAR into Vδ1 and Vδ2 γδ T cells, which significantly enhanced the tumor-specific cytotoxicity and migration ability of these cells. Additionally, CAR-Vδ2 T cells exhibited strong antigen presentation capabilities [65]. EP is a commonly used nonviral gene delivery method, particularly suitable for transient mRNA transfection. Schaft et al. applied EP to introduce gp100-specific T-cell receptors (TCR) or MCSP-specific CAR into γδ T cells, resulting in significant cytotoxicity against melanoma cells [66]. One advantage of mRNA transfection is its temporary CAR expression, which effectively reduces off-tumor toxicity, making it especially valuable for solid tumor research. The ‘Sleeping Beauty’ (SB) transposon system is an efficient and cost-effective nonviral gene editing method. The Cooper laboratory employed the SB transposon system to introduce CD19 CAR into γδ T cells and successfully achieved large-scale expansion [41]. The development of efficient and precise transfection strategies is essential for the successful application of CAR-γδ T cells in cancer therapy. Each strategy has its unique advantages, such as enhanced tumor-specific cytotoxicity, reduced off-tumor toxicity, as well as the potential to generate clinically applicable large-scale CAR-γδ T cell populations. These advancements bring CAR-γδ T cell therapies closer to clinical application, with promising results in both hematological and solid tumor treatments.

### Transduction approaches of CAR-M

The development of efficient gene delivery systems for macrophages requires careful consideration of their unique biological properties. Traditional adenoviral vectors exhibit limited efficacy due to macrophages' lack of coxsackievirus-adenovirus receptors, prompting the development of chimeric Ad5f35 vectors that exploit CD46-mediated entry [67-70]. These optimized vectors not only achieve efficient transduction but also confer additional benefits, including inflammasome activation that promotes M1 polarization and sustained CAR expression persisting over one month *in vitro* and 62 days *in vivo* [71]. Lentiviral and retroviral vectors can stably integrate exogenous genes into the host genome, achieving sustained gene expression. In murine bone marrow-derived macrophages (BMDM), HIV-1-derived lentiviruses can transduce effectively. However, in human myeloid cells, lentiviral transduction efficiency is inhibited due to the presence of SAMHD1, a myeloid-specific restriction factor [72,73]. To overcome this obstacle, researchers have introduced the Vpx accessory protein from HIV-2 or related simian immunodeficiency viruses, which induces SAMHD1 degradation, significantly improving lentiviral infection efficiency in macrophages [74]. This strategy not only enhances transgene delivery efficiency but also maintains the normal function of myeloid cells. Additionally, traditional retroviruses are typically unable to efficiently infect nonproliferating or low-proliferating cells. However, introducing a nuclear localization signal into the matrix protein of a C-type retrovirus markedly improves infection efficiency in macrophages [75]. Although not yet applied in CAR-M studies, these retroviral adaptations represent promising future tools.

Due to certain limitations in viral vectors regarding payload capacity and safety, several nonviral gene transfer strategies have been explored in recent years to enhance the modification efficiency and flexibility of CAR-M. Among these, the CRISPR/Cas9 system has emerged as an effective CAR delivery tool. Studies have shown that CRISPR/Cas9 can precisely insert an anti-GD2 CAR into the AAVS1 safe harbor site of human pluripotent stem cells (hPSCs), which then differentiate into M1 phenotype macrophages [76]. Moreover, transposon systems have been explored for gene modification in porcine aortic macrophages, allowing stable integration of transgenes into the host genome without relying on viral vectors [77]. In recent years, researchers have optimized mRNA modification strategies and improved transfection reagents to minimize toxicity and nonspecific activation in macrophages while enhancing CAR expression levels [78,79]. Additionally, the development of CpG demethylated plasmids helps avoid TLR9 recognition, thus achieving more sustained gene expression in RAW 264.7 macrophages and murine BMDM [80]. These research findings provide safe, flexible, and efficient alternatives for nonviral-mediated CAR-M cell modification.

**Figure 3:**
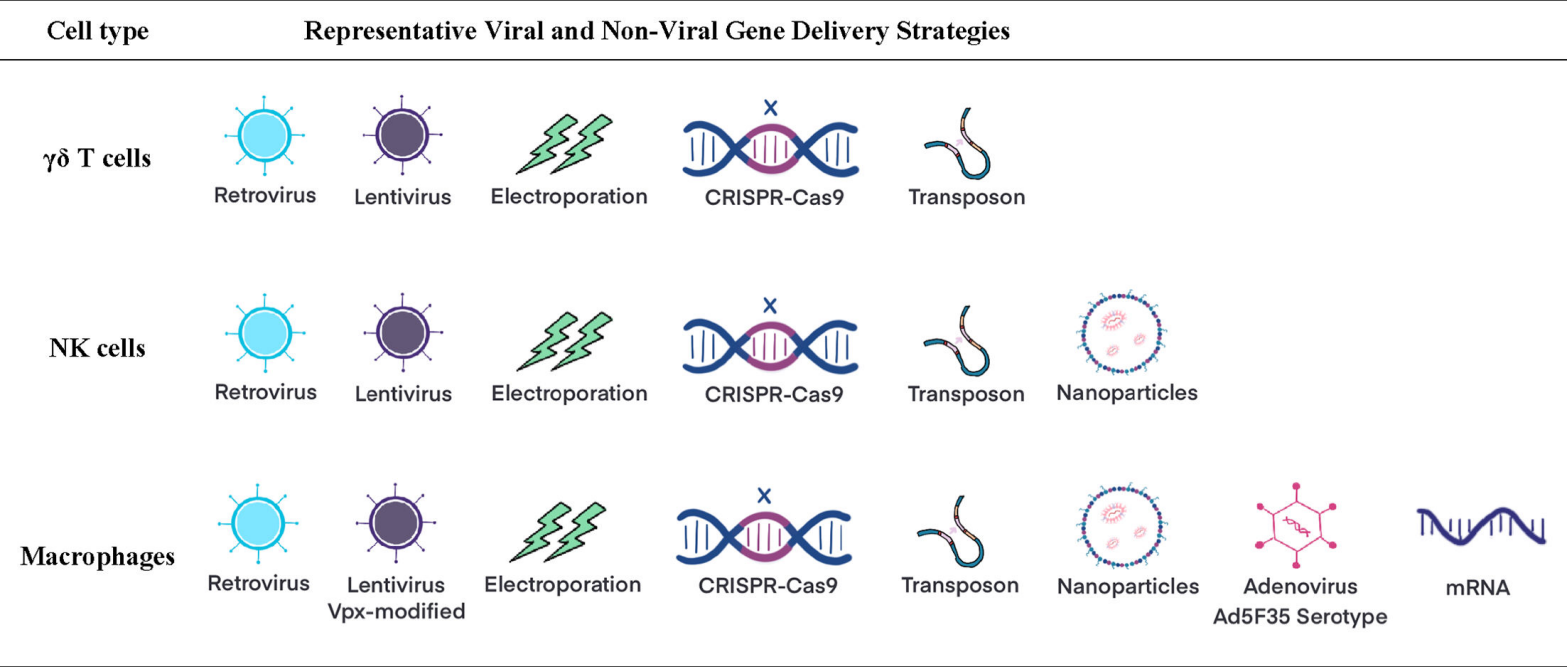
CAR gene delivery in NK, γδ T, and macrophages. Representative viral and nonviral methods for delivering CAR transgenes into NK cells, γδ T cells, and macrophages.

Distinct transduction strategies are tailored for NK cells, γδ T cells, and macrophages to accommodate their inherent biological differences. For CAR-NK cells, optimized lentiviral vectors work well, while mRNA electroporation, LNPs, and trogocytosis provide transient and flexible delivery. CRISPR/Cas9 and transposon systems further enhance CAR-NK functionality and persistence. In CAR-γδ T cells, both viral and nonviral approaches, including retroviral vectors, EP, and SB transposons, support efficient gene integration with advantages like reduced off-target toxicity and clinical scalability. CAR-M development faces transduction barriers, addressed by engineered adenoviral vectors and emerging nonviral tools such as CRISPR/Cas9, transposons, and optimized mRNA systems. These tailored transduction techniques enable efficient CAR expression, improved functionality, and greater clinical applicability across diverse immune cell platforms (Figure 3).

### Limitations and future directions

Despite advances in CAR transduction techniques, each immune cell type faces significant challenges. CAR-NK cells exhibit generally low viral transduction efficiency, while nonviral methods such as mRNA electroporation provide transient expression, limiting sustained efficacy. Efficient expansion, purification, and stable transduction of γδ T cell subsets remain technically challenging, with notable differences in efficiency among subpopulations. CAR-M, being terminally differentiated, are difficult to genetically modify stably; most approaches rely on transient expression or progenitor cell reprogramming, raising concerns about scalability and clinical feasibility. Future efforts should optimize nonviral platforms, enhance transduction efficiency while preserving cell functionality, and establish clinical-grade, scalable production processes.

## Distinct antitumor mechanisms of CAR-engineered immune cells

The three major CAR-modified immune cell types exhibit unique therapeutic mechanisms and efficacy profiles that reflect their distinct biological properties. CAR-NK cells integrate CAR targeting with NK cells’ intrinsic MHC-unrestricted cytotoxicity. They offer reduced toxicity, immediate allogeneic availability, and self-limiting persistence, though their therapeutic duration is limited by low proliferation. CAR-γδ T cells represent a hybrid platform that merges the antigen recognition versatility of γδ TCRs with CAR-directed specificity, enabling both MHC-independent tumor targeting and adaptive immune functions. Meanwhile, CAR-M leverage their natural tumor tropism and plasticity to execute multi-pronged antitumor activity through direct phagocytosis, antigen presentation, and dynamic microenvironment remodeling—capabilities that position them as particularly promising for solid tumor applications. Together, these platforms demonstrate how different immune cell lineages can be strategically harnessed through CAR engineering to create complementary therapeutic approaches with distinct mechanistic advantages.

### CAR-NK

NK cells, representing 10–20% of peripheral blood (PB) lymphocytes, are derived from bone marrow CD34^+^ lymphoid progenitor cells and serve as crucial effectors in innate immune surveillance and antitumor responses [81,82]. While functionally analogous to CD8^+^ cytotoxic T cells in their target cell elimination mechanisms, NK cells are distinguished by their innate recognition capacity, operating independently of somatic recombination and antigen-specific TCRs [83]. NK cells exhibit unique cytotoxic properties that distinguish them from T cells, making them particularly promising for immunotherapy applications. Unlike T cells that require antigen sensitization, NK cells demonstrate MHC-unrestricted cytotoxicity capable of directly eliminating tumor cells. This innate cytotoxic capacity is governed by a finely tuned balance between germline-encoded activating and inhibitory receptors, with the net cytotoxic response determined by the integration of these opposing signals. The combination of these features—including immediate tumor recognition, nonspecific cytotoxicity, and receptor-mediated response modulation—establishes NK cells as an exceptionally promising platform for cellular immunotherapy [84].

CAR-NK cells merge CAR-mediated specificity with NK cells’ innate MHC-independent cytotoxicity, offering advantages over conventional CAR-T therapies: [1] Limited lifespan: CAR-NK cells have a finite lifespan, decreasing the risk of persistent attacks on normal tissues [85] [2]. Reduced toxicities: CAR-NK cell therapy is associated with a lower likelihood of inducing CRS and neurotoxicity [86] [3]. Allogeneic use: Lack of graft-versus-host disease (GvHD) after CAR-NK infusion enables ‘off-the-shelf’ product generation and cost reduction [87] [4]. Abundant sources: NK cells are abundant in clinical samples, sourced from PB, umbilical cord blood (UCB), induced pluripotent stem cells (iPSCs), or even the NK-92 cell line, as outlined in Table 1, considering the source of NK cells, their pros and cons, and ongoing clinical research [88] [5]. Multiple mechanisms: Besides CAR-dependent targeted killing, CAR-NK cells retain the ability to recognize tumor cells with target loss via CAR-independent mechanisms [89]. When activated, NK cells deploy diverse cytotoxic strategies: perforin and granzymes are released to promote apoptosis in target cells [90], mediate antibody-dependent cellular cytotoxicity (ADCC) via CD16, and secrete cytokines including IFN-γ and TNF-α to exert antitumor effects, remodel the TME, and promote adaptive immune responses [91]. Unlike T cells, NK cells can respond immediately without prior antigen sensitization and recognize tumor cells with down-regulated MHC-I, preventing immune evasion. Nevertheless, challenges remain for CAR-NK therapy, including limited persistence and proliferative capacity, as well as the necessity of careful target antigen selection to maximize efficacy and reduce off-target effects. Ongoing studies seek to address these limitations to fully realize its clinical potential.

**Table 1:** CAR-NK Cells from different sources

NK cell type	Advantages	Disadvantages	Targets	Clinical trial identifier
PB-NK	Mature and highly cytotoxic	Difficult expansion	Nkg2dl	NCT04623944
	Autologous cells reduce risk of immune rejection	Potential pathogen exposure	Mesothelin	NCT03692637
	Donor is readily available	Cell manufacturing are complex and expensive	Claudin6	NCT05410717
		Non homogenous	CD19	NCT05020678
			CD19	NCT00995137
			Nkg2dl	NCT03415100
CB-NK	High proliferation capacity	Immature cells，may have reduced function compared to adult cells	CD19	NCT04796675
	Rich in sources	Reduced function compared to primary NK cells	CD19	NCT03056339
	Ease of collection	Difficult expansion	CD19	NCT05472558
			CD19	NCT04796688
			BCMA	NCT05008536
			Nkg2dl	NCT05247957
			CD5	NCT05110742
NK92	Easy to expand and to engineer;	Requires irradiation	HER2	NCT03383978
	Homogenous product;	Lacks CD16	CD33	NCT02944162
	Off the shelf product		NKG2D	NCT05528341
			PD-L1	NCT04390399
			PD-L1	NCT04847466
iPSC-NK	Does not require irradiation	Immature phenotype	CD19	NCT05336409
	Large cell doses	Complex and expensive	CD19/CD20	NCT04245722
	Homogenous product		Mica/MICB	NCT05395052
			BCMA	NCT05182073

### CAR-γδ T

T cells are classified into two principal subsets according to TCR type: αβ T cells (such as CD4, CD8) and γδ T cells. In the human PB lymphocytes, αβ T cells represent the predominant subset, while γδ T cells usually make up only 1%–10% of the total T-cell population. The latter are primarily found in epithelial and mucosal tissues [92]. γδ T cells can directly bind to antigens without requiring MHC molecules for antigen presentation. This distinctive feature makes γδ T cells a promising CAR-T cell family subset. The pathways through which CAR-γδ T cells drive antitumor responses include (1) Innate Immunity: CAR-γδ T cells recognize and eliminate tumor cells via recognition of various surface antigens on the tumor cells, relying on innate immune responses. (2) Adaptive Immunity: CAR-γδ T cells are also capable of mediating antitumor effects via their γδ TCRs, which allows for adaptive immune responses against tumors. (3) CAR-Mediated Anti-Tumor Activity: CARs expressed on γδ T cells further enhance their antitumor activity by providing an additional mechanism for targeting and killing cancer cells. Due to their unique tumor recognition capabilities, research is under way to investigate whether CAR-γδ T cells can be utilized as effective effectors in adoptive cell immunotherapy for cancer [93]. Human γδ T cells express a distinct T cell receptor composed of γ and δ chains. The δ chain diversity is determined by three Vδ genes (Vδ1, Vδ2, and Vδ3), which classify human γδ T cells into three major subsets. These subsets—particularly Vδ1 and Vδ2 T cells, along with polyclonal γδ T cell populations—serve as primary cellular sources for developing CAR-engineered γδ T cell therapies.

### CAR-M

**Figure 4:**
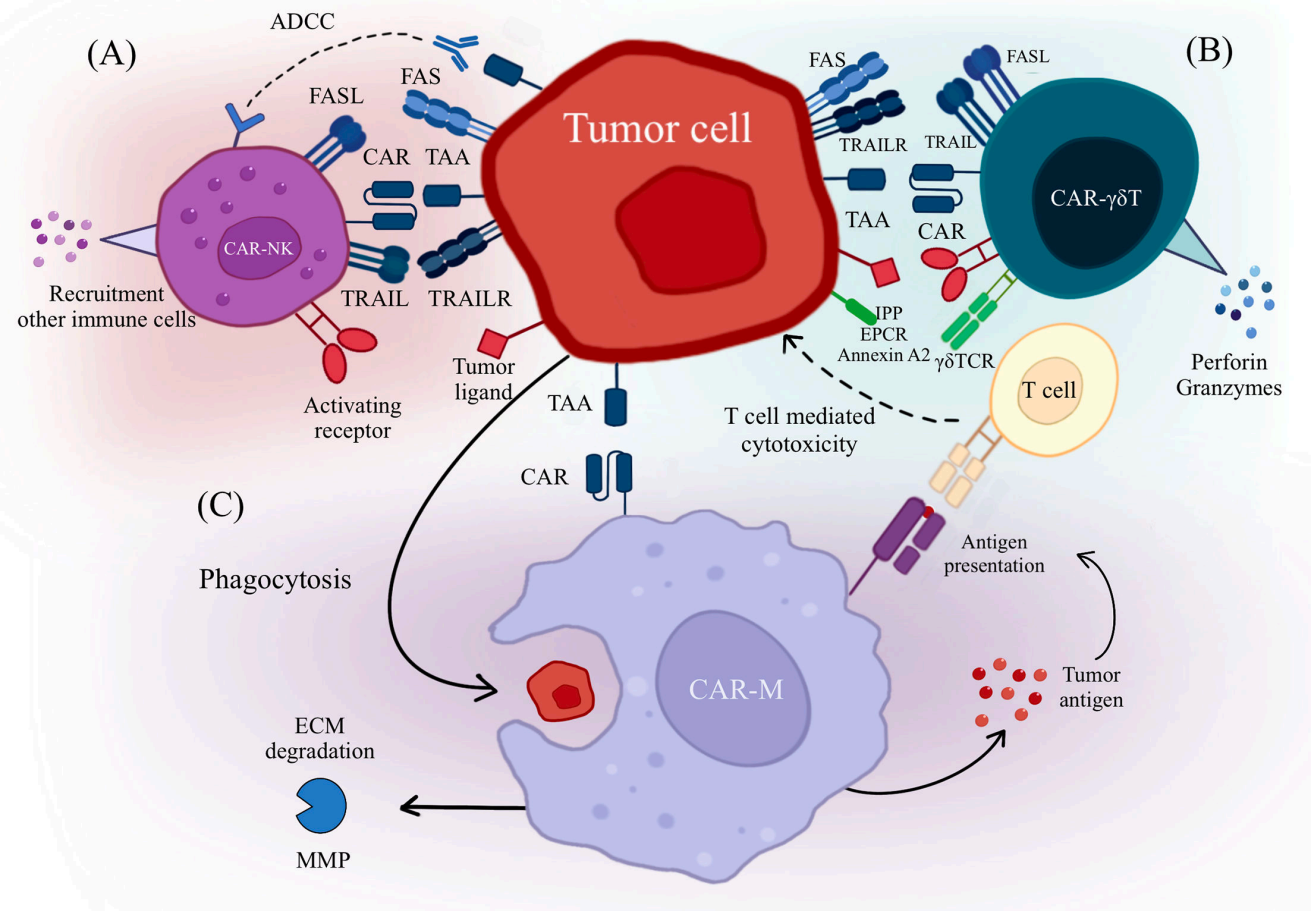
Cytotoxic mechanisms of CAR-engineered immune cells. (**A**) CAR-NK cells recognize cancer cells through their CAR and activating receptors. They exert antitumor effects by engaging death receptors such as FasL and TRAIL, participating in ADCC, and secreting cytokines to activate other immune cells. (**B**) CAR-γδ T cells recognize tumor cells not only through their CAR but also via their γδTCR and co-receptors. They mediate antitumor activity through the TRAIL and Fas/FasL pathways, as well as by secreting granzyme B and perforin to induce tumor cell apoptosis. (**C**) CAR-M penetrate solid tumors by secreting MMPs to degrade the extracellular matrix. They recognize tumor cells via their CAR, directly eliminate them through phagocytosis, and further enhance antitumor immunity by presenting tumor antigens to activate T cells. ADCC, antibody-dependent cellular cytotoxicity; CAR，chimeric antigen receptor; CAR-M，CAR-macrophages; ECM, extracellular matrix; EPCR, endothelial protein C receptor; FAS, first apoptosis signal receptor; FASL, Fas ligand; IPP, isopentenyl pyrophosphate; MMP, matrix metalloproteinases; TAA, tumor-associated antigen; TRAIL, tumor necrosis factor-related apoptosis-inducing ligand; TRAILR, TRAIL receptor.

Unlike T cells, macrophages are naturally present in the TME and represent the most abundant infiltrating immune cells within the TME [94]. Macrophages, as innate immune cells, exert antitumor effects through various mechanisms, including active trafficking to tumor sites, direct phagocytosis of tumor cells, modulation of the TME, and antigen presentation [67,95]. Furthermore, macrophages’ plasticity allows them to adapt to their surroundings readily. Given the abundance of macrophages in various solid tumor TMEs and their adaptability, numerous studies have explored the modification of macrophages with CARs as a strategy to address obstacles in CAR-T cell therapy (Figure 4).

### Limitations and future directions

CAR-engineered NK cells, γδ T cells, and macrophages each exhibit distinct antitumor mechanisms, yet the relative contribution of CAR-dependent versus CAR-independent pathways remains unclear. CAR-NK cell signaling integration in immunosuppressive TME is not fully elucidated. In CAR-γδ T cells, potential synergy between CAR-mediated recognition and endogenous γδ TCR activity, as well as functional exhaustion during chronic antigen exposure, requires further study. CAR-macrophages demonstrate strong tumor infiltration and phagocytic activity, but evidence regarding long-term survival, antigen presentation capacity, and risks of excessive inflammation remains limited. Future studies should leverage *in vivo* models to dissect these mechanisms, clarify how CAR engineering reshapes intrinsic effector programs, and develop strategies to enhance efficacy while minimizing off-target effects or immune dysfunction.

## Clinical translation status of CAR-engineered immune cell therapies

The clinical development of CAR-engineered immune cell therapies has progressed along distinct trajectories for NK cells, γδ T cells, and macrophages, each demonstrating unique therapeutic advantages. CAR-NK cell therapy integrates targeted specificity with innate MHC-independent killing, showing encouraging efficacy but limited persistence. CAR-γδ T cells link innate and adaptive immunity, exhibiting efficacy against both hematologic and solid tumors through CD20 and CD123 targeting, despite facing microenvironmental barriers in solid tumor applications. The most nascent of these approaches, CAR-M therapy, demonstrates compelling preclinical potential for solid tumors via HER2-directed phagocytosis and TME modulation. These complementary platforms collectively advance the field of cellular immunotherapy, with ongoing research focused on overcoming limitations in cell persistence, tumor infiltration, and immunosuppression to unlock their full clinical potential.

### Clinical translation of CAR-NK

CAR-NK cell therapy is currently under active clinical investigation, with 38 registered trials underway. However, the sample sizes of existing CAR-NK clinical studies remain limited, as most trials remain at the initial phase I/II stage. To date, the number of enrolled patients generally ranges from single digits to a little over thirty, thereby constraining the generalizability of the findings and reducing statistical power. One of the largest single-center trials was conducted at MD Anderson Cancer Center by Marin et al., in which 37 patients were enrolled. In this study, cord blood–originated anti-CD19 CAR-NK cells induced responses in 8 of 11 (73%) patients with relapsed/refractory CD19^+^ hematologic malignancies, including 7 complete remissions (CR). Notably, the infused cells showed sustained persistence for over 12 months despite limited expansion [86]. The allogeneic peripheral blood-derived CAR-NK product NKX019 also showed promising efficacy in an initial cohort of 19 patients, with an objective response rate (ORR) of 80% and CR rate of 70% in high-dose NHL patients; several patients who relapsed could achieve CR upon re-treatment. The iPSC-derived CAR-NK product FT596 demonstrated similar efficacy in 20 patients, with an ORR of 73% and CR of 64% at single doses ≥9 default 10⁷ cells, including responses in patients previously treated with CAR-T therapy [96]. Another iPSC-derived, multiengineered CAR-NK product, CNTY-101, showed an ORR of 40% in 12 patients, with durable CR observed even at lower doses [97]. In terms of safety, CAR-NK treatment showed favorable tolerability. Most trials reported no dose-limiting toxicities (DLTs), immune effector cell-associated neurotoxicity syndrome (ICANS), or GvHD. Only a few grade 1–2 CRS events were reported, and no severe adverse events were observed. Importantly, allogeneic CAR-NK cells did not exhibit significant rejection, highlighting their potential safety advantage in allogeneic settings. Overall, although the current clinical trials are limited in sample size, preliminary results suggest that CAR-NK cell therapy can achieve high response rates in B-cell malignancies while maintaining favorable safety and the potential for repeat dosing. These features support its clinical application in hematologic malignancies and other indications and underscore the need for further optimization of dosing, cell sources, and administration strategies. In addition to CD19-targeted therapies, CAR-NK cell trials targeting CD33 for AML involved relatively small sample sizes (3–10 patients) [98,99]. The response rate reached up to 60% in some studies (MRD-CR), but efficacy varied considerably, with some patients relapsing shortly after an initial response. Overall, the safety profile was favorable, with mostly mild CRS (grade 1–2) reported and no cases of ICANS or GvHD. While these results emphasize the therapeutic potential of CAR-NK cells, key challenges remain regarding their limited *in vivo* persistence and expansion capacity—critical factors requiring optimization for broader clinical application.

Beyond hematologic malignancies, CAR-NK cells show distinct advantages for solid tumor therapy, including: [1] potent MHC-unrestricted cytotoxicity [2], intrinsic tumor-homing capability, and [3] improved safety relative to CAR-T cells. Preclinical studies have established their potent activity across various solid tumors [100,101], and ongoing phase I/II clinical trials have provided preliminary evidence of feasibility and therapeutic potential [102]. For example, in an NKG2D ligand-targeting CAR-NK cell trial [32], three patients with colorectal cancer received intraperitoneal CAR-NK cell treatment. Autologous CAR-NK cells were administered to one patient, and haploidentical allogeneic CAR-NK cells were given to the other two. Two patients showed stable disease with no progression of peritoneal lesions on CT imaging, and one patient exhibited marked tumor shrinkage in the liver after multiple rounds of dose-escalated infusions. The therapy was well tolerated, with no reported ICANS, GvHD, or dose-limiting toxicities, and the treatment was associated with no CRS above grade 1. In clinical trials targeting PD-L1, PD-L1 t-haNK, an NK-92–derived product, was assessed in a phase I study enrolling nine patients with locally advanced or metastatic solid tumors. Four patients achieved stable disease (SD), and no occurrences of CRS, ICANS, or GvHD were reported, and the majority of adverse events were grade 1–2 [103]. In a subsequent phase II study, 83 patients with pancreatic cancer were treated with PD-L1 t-haNK. The median overall survival (OS) was 5.7 months, and the median progression-free survival (PFS) was 2.3 months, with 37% of patients maintaining SD for more than 8 weeks. The therapy exhibited good tolerability, and no severe CRS, ICANS, or GvHD were detected [104]. Other CAR-NK studies have targeted antigens such as BCMA [105], chimeric costimulatory converting receptor (CCCR) [106], PSMA [107], and HER2 [108]. Overall, these studies involved small sample sizes and demonstrated good treatment tolerability. Except for patients receiving CCCR-NK92, who experienced severe CRS, most other studies reported no severe CRS, ICANS, or GvHD events. However, clinical experience remains substantially more limited for solid tumors than for hematologic malignancies, necessitating further validation.

Although CAR-NK cells have shown good tolerability and certain efficacy in multiple clinical trials, notable limitations remain. First, most studies have small sample sizes and are early-phase I/II trials, limiting the generalizability and statistical robustness of the findings. Second, several studies report rapid relapse after initial responses, indicating limited *in vivo* survival and proliferation of CAR-NK cells. Third, in solid tumors, although some patients achieved stable disease or partial responses, overall efficacy is lower than in hematologic malignancies, likely resulting from the immunosuppressive TME and barriers to NK cell penetration. In addition, dose appears to significantly affect efficacy, and while repeated dosing shows potential, it lacks systematic validation. In summary, despite the high tolerability, flexible administration, and feasibility of multiple infusions, the clinical application of CAR-NK cells requires larger-scale, long-term studies to further validate efficacy and explore strategies to optimize cell sources, manufacturing processes, and enhance persistence and antitumor activity.

### Clinical translation of CAR-γδ T

CAR-engineered γδ T cells represent a potential and innovative strategy for cancer immunotherapy, demonstrating significant potential across both hematologic malignancies and solid tumors. As a therapy for hematologic malignancies, CD20-targeted CAR-γδ T cells showed promising therapeutic effects. ADI-001, an allogeneic Vδ1 CAR T cell directed against CD20, is presently undergoing clinical evaluation in patients with B-cell malignancies. In at least 24 enrolled patients, preliminary results demonstrated an ORR of 71% and a CR rate of 63%, with no observed GvHD [109]. However, given the early-stage nature of the study, limited follow-up duration, and the absence of long-term safety and durability data, further large-scale clinical studies are needed to validate the benefits and safety. Additionally, CD123-targeted CAR-γδ T cells have shown promise in treating acute myeloid leukemia (AML). In preclinical studies, CD123 CAR-γδ T cells effectively suppressed tumor growth in mouse models and enhanced survival [110]. These findings provide a solid foundation for the application of CAR-γδ T cell therapy in hematologic malignancies.

The application in solid tumors, while more challenging because of immunosuppressive microenvironments and infiltration barriers, has shown equally compelling preclinical results. Adicet Bio’s GPC3-targeted CAR-Vδ1 T cell therapy (ADI-002) demonstrated potent tumor control and survival extension in hepatocellular carcinoma models through combined CAR targeting and IL-15 secretion [111]. Another significant advance involves Claudin18.2-specific CAR-γδ T cells, which effectively targeted this pan-cancer antigen in gastric cancer models, overcoming microenvironmental suppression with superior cytotoxicity over classical l CAR-αβ T cells [112]. Recently, a first-in-human phase I trial (NCT06018363) further explored CAR-γδ T cells in central nervous system tumors. The study investigated intrathecal infusion of allogeneic B7H3 CAR-γδ T cells (QH104) in patients with recurrent glioblastoma [113]. Seven patients participated in the study, and the median follow-up period was 6.5 months. QH104 demonstrated a favorable safety profile, with no dose-limiting toxicities, ≥ grade 3 CRS, ICANS, or GvHD reported. Fever and headache were the most frequent adverse events, along with elevated IL-6 and IFN-γ in the cerebrospinal fluid. Clinically, the ORR was 42.9% (3/7), with a disease control rate of 100% (7/7). Persistence of infused cells was confirmed beyond 30 days by FACS and qPCR, and preliminary analysis suggested a positive correlation between clinical response and B7H3 expression levels. These findings offer initial clinical evidence indicating that CAR-γδ T cells are safe and potentially effective in CNS malignancies, though validation in larger cohorts is required. These findings are driving rapid clinical translation, with 8 active CAR-γδ T cell clinical trials currently underway (Table 2), reflecting growing recognition of their therapeutic potential across diverse malignancies. Collectively, CAR-γδ T cell therapy has demonstrated encouraging safety and early efficacy signals in both hematologic malignancies and solid tumors. Nonetheless, most clinical studies remain in their early phases, constrained by small sample sizes and short follow-up durations. Future large-scale, long-term studies are essential to comprehensively validate their safety and durability, while optimization of cell sources, manufacturing processes, and functional enhancements will be essential for promoting their broader clinical application.

**Table 2:** Ongoing clinical trials of CAR-γδT cell therapy

NCT number	Start date	Target	Conditions	Dosage/frequency	Phase/stage	Academia/company
NCT04107142	2019/12/01	NKG2DL	Colorectal cancer, triple-negative breast cancer, sarcoma, nasopharyngeal carcinoma, prostate cancer, gastric cancer	3 × 10^8^, 1 × 10^9^, 3 × 10^9^(Each cycle of therapy will consist of 4 intravenous infusions, given 7 days apart)	Phase I/ Not yet recruiting	CytoMed Therapeutics Pte Ltd.
NCT04702841	2020/06/03	CD7	Relapsed and refractory CD7 positive T cell-derived malignant tumors	0.2–5 × 10^6^/kg (once)	Phase 1/ Recruiting	PersonGen BioTherapeutics (Suzhou) Co., Ltd.|Anhui Provincial Hospital
NCT05302037	2022/04/01	NKG2DL	Advanced solid tumors or hematological malignancies	1 × 10^7^, 1 × 10^8^, 3 × 10^8^ or 1 × 10^9^ per infusion at an interval of one infusion every 7 days	Phase I / Not yet recruiting	CytoMed Therapeutics Pte Ltd|National University Hospital, Singapore
NCT02656147	2017/10/01	CD19	Relapsed or refractory B cell-derived acute lymphoblastic leukemia (ALL), chronic lymphoblastic leukemia (CLL), and non-Hodgkin lymphoma	Unknown	Phase I/ Unknown status	Beijing Doing Biomedical Co., Ltd.
NCT04165941	2020/02/11	Unknown	Glioblastoma multiforme (GBM)	Unknown	Phase I/recruiting	University of Alabama at Birmingham IN8bio, Inc, United States
NCT04796441	2020/12/16	Unknown	Patients with relapsed AML after transplantation	Unknown	Phase I/recruiting	Hebei Senlang Biotechnology Inc., Ltd. China
NCT04735471	2021/03/04	CD20	R/R B cell lymphoma	3 × 10^7^, 1 × 10^8^, 3 × 10^8^ or 1 × 10^9^	Phase I/recruiting	Adicet Bio, Inc, United States

### Clinical translation of CAR-M

To date, researches involving CAR-M have primarily been in the preclinical stages, with a growing number of preclinical studies targeting solid tumors including hepatocellular carcinoma [114], gastric cancer [115], breast cancer [116], and others. Dong et al. developed HER2-CAR macrophages based on human peritoneal macrophages for gastric cancer treatment. In a mouse model of peritoneal carcinomatosis with HER2-positive gastric cancer, intraperitoneal injection of CAR macrophages not only demonstrated significant antitumor efficacy but also showed good safety. Once activated, CAR macrophages adopted an M1 phenotype, enhancing antigen-specific phagocytosis and presentation, thereby promoting T cell expansion and further enhancing antitumor immune responses [115]. Currently, four CAR-M–based clinical trials are registered on ClinicalTrials.gov. One trial, targeting HER2-overexpressing solid tumors, is designed to evaluate the efficacy of autologous HER2-targeted CAR-M delivered via adenoviral transduction (CT-0508). This trial utilizes the CAR-M platform established by Klichinsky et al., employing a chimeric adenoviral vector Ad5f35 carrying a CAR with a HER2-specific scFv to induce macrophage differentiation into a pro-inflammatory M1-like phenotype. Fourteen patients were enrolled, and among those with HER2 3 + tumors, 44% achieved stable disease at 8 weeks post-treatment. In terms of safety, no DLTs, severe CRS, or ICANS were observed [117]. Building on the experience with CT-0508, its iterative product, HER2-targeted CAR-monocyte therapy (CT-0525), is undergoing a phase I clinical trial involving six patients to assess safety, immunogenicity, pharmacokinetics, and related biomarkers [118]. Compared with CT-0508, CT-0525 is expected to demonstrate improved clinical antitumor activity, with the potential for higher dosing, enhanced tumor infiltration, prolonged persistence, and reduced manufacturing time. Another trial (NCT03608618) is investigating CAR-M expressing a mesothelin-targeting CAR (MCY-M11) for ovarian cancer and peritoneal mesothelioma and is currently recruiting [119]. A third trial (NCT06224738), initiated in 2024, is assessing the safety and therapeutic potential of CAR-M (MAC-001) in patients with advanced HER2-positive gastric cancer. Additionally, a single-center, single-arm, dose-escalation exploratory trial (NCT06562647) is testing mesothelin-CAR-M (SY001) for safety, tolerability, pharmacokinetics, and preliminary efficacy in advanced solid tumors. These clinical trials and other pioneering studies represent critical steps toward the clinical translation of CAR-M in cancer therapy. Importantly, most trials have small sample sizes, limited follow-up, and lack long-term safety and durability data, while the mechanisms of action and optimal administration strategies for different CAR-M products remain unclear. Therefore, despite showing promising preliminary safety and potential efficacy, the clinical application of CAR-M requires larger-scale, long-term studies to validate efficacy, optimize dosing, cell sources, and strategies for modulating the TME.

### Limitations and future directions

Compared with CAR-T therapies, clinical progress of CAR-NK, CAR-γδ T, and CAR-macrophage platforms is still in its infancy. CAR-NK cells exhibit favorable safety but limited persistence and efficacy, indicating the need for strategies to enhance long-term responses. Clinical data for CAR-γδ T cells remain scarce, and it is uncertain whether early-stage findings will translate into consistent clinical performance. CAR-macrophages have only undergone first-in-human trials, with limited efficacy data and safety concerns related to inflammatory responses. Current clinical trials are generally small in scale with short follow-up periods, lacking long-term safety and durability assessments. Regarding clinical application, CAR-NK cells possess unique advantages owing to their MHC-independent cytotoxicity and minimal potential for GvHD, yet limitations exist in manufacturing scale, *in vivo* persistence, and tumor penetration; clinical use may prioritize patients with high safety requirements and suitability for allogeneic therapy. γδT cells bridge innate and adaptive immunity and may be suitable for hematologic malignancies and certain solid tumors, but challenges in cellular heterogeneity and expansion persistence must be addressed; clinical decisions should consider specific pathological features and the selection of cell subsets. CAR-M cells exhibit strong tumor tropism and capacity for TME remodeling, underscoring their therapeutic potential in poorly infiltrated solid tumors, but potential inflammatory activation and long-term survival issues require close monitoring. Future research should integrate early clinical trial data, biomarker-based patient stratification, and direct comparisons across different CAR platforms to guide clinical selection and optimize personalized treatment strategies, ultimately enabling next-generation immune cell therapies to overcome the challenges of traditional CAR-T in terms of safety and durability.

## Challenges and optimization strategies for CAR-engineered immune cell therapies

While CAR-NK, CAR-γδ T, and CAR-M therapies demonstrate considerable promise in cancer immunotherapy, each platform faces distinct biological and technical hurdles requiring tailored solutions. For CAR-NK cells, the primary challenges center on enhancing expansion efficiency, cytotoxic potency, and *in vivo* persistence, with current strategies focusing on cytokine supplementation (e.g. IL-15) and genetic modifications to activation pathways. CAR-γδ T cell therapy contends with expansion difficulties in immunodeficient settings and microenvironmental suppression, addressed through cytokine-supported propagation and immune checkpoint modulation. The emerging CAR-M platform, despite its impressive phagocytic capacity, must overcome limitations in sustained antitumor activity and microenvironmental resistance, driving innovations in macrophage polarization and survival engineering. Across all three platforms, researchers use advanced genetic engineering, cytokine network optimization, and microenvironment adaptation to balance therapeutic efficacy and safety. These concerted efforts continue to advance these promising immunotherapies toward broader clinical application.

### CAR-NK

#### Enhancing the expansion efficiency of CAR-NK cells

Enhancing the expansion capacity of CAR-NK cells remains a critical challenge in the field. Recent studies have identified several promising approaches to improve NK cell proliferation. The use of irradiated feeder cells expressing membrane-bound IL-15 or IL-21 in combination with 4–1BBL has been demonstrated to significantly boost NK cell expansion rates [120,121]. Particularly noteworthy is the human B lymphoblastoid cell line 721.221 expressing membrane-bound IL-21 (mIL-21), which exhibits greater NK cell expansion potential and stronger cytotoxic activity than conventional K562.mbIL-21 feeder systems [122]. Another effective strategy involves the co-expression of IL-15 with its receptor IL-15Rα, which has proven particularly beneficial for augmenting the proliferation of NK-92 cells [39]. These findings collectively highlight the importance of cytokine signaling optimization in developing more robust CAR-NK cell expansion protocols.

#### Enhancing the cytotoxic function of CAR-NK cells

Overcoming the insufficient persistence and cytotoxicity of CAR-NK cells represents a crucial challenge for their clinical translation. Recent advances have identified several promising strategies to enhance their therapeutic potential, including genetic engineering, cytokine stimulation, and novel agonists. Genetic engineering approaches have shown particular promise, with IL-15-secreting CD19 CAR UCB NK cells demonstrating improved durability and cytotoxicity in phase I/II clinical trials [123]. The combination of TcBuster transposon system with Epstein–Barr virus-transformed lymphoblastoid feeder cells, coupled with CRISPR/Cas9-mediated SH2 protein knockout, has successfully generated highly cytotoxic CLL-1-targeting CAR-NK cells [62]. Cytokine-based strategies have also proven effective. The generation of cytokine-induced memory-like (CIML) NK cells through IL-12/IL-18 preactivation enhances antitumor activity, suggesting their potential as superior platforms for CAR-NK cell production [124]. Memory-like NK cells engineered with anti-EphA2 CAR demonstrated elevated IFN-γ and TNF production along with improved cytotoxicity against head and neck squamous cell carcinoma [125]. STING pathway activation represents another innovative approach. The STING agonist ADU-S100 enhances both migratory capacity and cytotoxicity of mesothelin-targeting CAR-NK cells [126], while cGAMP synergizes with mesothelin-specific CAR-NK-92 cells to significantly improve tumor suppression and therapeutic outcomes in pancreatic cancer models [127]. These findings collectively highlight the multifaceted strategies being employed to optimize CAR-NK cell function, ranging from genetic modifications to cytokine programming and innate immune activation, paving the way for more potent CAR-NK cell therapies.

#### Enhancing the safety of CAR-NK cell therapy

CAR-NK cell therapy demonstrates superior advantages compared with CAR-T cell therapy, particularly with regard to GvHD and CRS, as evidenced by clinical trials showing no GVHD occurrence in 11 HLA-mismatched patients treated with cord blood-derived CAR-NK cells [86]. The superior safety is linked to cytokine differences. CAR-NK cells mainly produce IFN-γ and GM-CSF, while CAR-T cells release inflammatory cytokines such as TNF-α, IL-1β, IL-2, and IL-6 that drive CRS [128,129]. However, instances of CRS have been observed in CAR-NK treatments, as evidenced by a case involving nonsmall cell lung cancer patients who received chimeric costimulatory converting receptor-NK-92 cells (CCCR-NK-92). During treatment, these patients experienced hypotension, shock, high fever, hemoptysis, myalgia, liver injury, and coagulation abnormalities, accompanied by a tenfold up-regulation of IFN-γ and TNF-α. The underlying cause of this adverse reaction was hypothesized by researchers to potentially stem from the construction of CCCR, which encompasses PD-1-NKG2D-41BB. This approach converts the inhibitory PD-1 signaling into an activating pathway upon tumor cell killing. However, such an approach might lead to excessive immune cell activation, thereby triggering the increased production of cytokines such as TNF-α [106]. When applying CAR-NK cell therapy, monitoring biomarker changes that predict the risk of CRS is essential. If abnormalities are detected, early intervention should be administered promptly to prevent severe adverse reactions in patients. Additionally, clinical practitioners should work to minimize tumor burden before CAR-NK cell infusion. It is important to pay attention to the number of CAR-NK cells infused, gradually increasing the cell dose after ensuring the patient’s condition is stable, thereby reducing the incidence of CRS.

### CAR-γδ T

CAR-γδ T cell therapy has gained attention as a promising modality, garnering considerable research interest due to its dual advantages of potent antitumor activity and favorable safety profile. While preclinical studies and early clinical trials have demonstrated its therapeutic potential, significant obstacles still need to be overcome to enable wider clinical application.

#### Promoting γδ T cell expansion and persistence

A primary challenge in CAR-γδ T cell therapy is achieving sufficient cell expansion and persistence, particularly in immunodeficient environments where the absence of IL-15 severely compromises γδ T cell survival and proliferation. To overcome this limitation, researchers have successfully employed IL-15 supplementation, which has demonstrated dual benefits in preclinical models: [1] significantly improving γδ T cell persistence and [2] maintaining robust antitumor activity even upon repeated tumor exposure. This approach has shown particular promise in AML models, where IL-15-enhanced CAR-γδ T cells exhibited sustained therapeutic efficacy [110,130]. The cytokine’s ability to simultaneously support proliferation and preserve cytotoxic function positions IL-15 as a pivotal factor in optimizing CAR-γδ T cell therapies.

#### Overcoming the immunosuppressive TME

The therapeutic potential of γδ T cells in solid tumors is significantly constrained by the immunosuppressive TME, where factors like hypoxia and inhibitory signals impair their functionality. To address this limitation, current research focuses on two key optimization strategies: microenvironmental adaptation and exhaustion prevention [42,43]. First, bispecific T-cell engagers (BiTE) targeting both HLA-G and PD-L1 have shown promise in overcoming immune checkpoint-mediated suppression, effectively eliminating dual-positive solid tumors [131]. Second, resembling conventional T cells, γδ T cells are susceptible to exhaustion from chronic antigen exposure [132], with emerging evidence suggesting TGF-β1 may play a paradoxical role in maintaining their antitumor capacity. While TGF-β is known to inhibit mTOR signaling and induce metabolic quiescence in T cells [133], studies demonstrate that TGF-β1 treatment can paradoxically boost the cytotoxic activity of Vγ9Vδ2 T cells, potentially by mitigating exhaustion pathways [134]. These findings highlight the complex interplay between suppressive factors and γδ T cell function in the TME, underscoring the need for multifactorial approaches to optimize their therapeutic performance.

#### Selecting γδ T cells with better antitumor function

The functional diversity among γδ T cell subsets presents both opportunities and challenges for their therapeutic application, with distinct subsets exhibiting markedly different—and sometimes opposing—roles in tumor immunity. Research has indicated that Vδ1 T cells might have an immunosuppressive role in ovarian, gallbladder, and colorectal cancer, potentially promoting tumor progression [135-138]. Conversely, other studies suggest that Vδ1 T cells can exert antitumor effects and are associated with favorable patient outcomes [139-141]. A recent study suggests that γδ T cells can be a double-edged sword: they play a suppressive role in early-stage tumors but change their stance and promote tumor growth as the tumor progresses. These opposing functional characteristics are related to the specific subgroups of γδ T cells: antitumor γδ T cell subgroups resident in intestinal epithelium are mainly Vγ1^+^ and Vγ7^+^ cells, while pro-tumor subgroups infiltrating tumors are mainly Vγ4^+^ and Vγ6^+^ cells [142]. Therefore, further research into the tumor treatment mechanisms of γδ T cells is needed to prevent adverse effects during the application of CAR-γδ T cell therapy.

#### Enhancing γδ T cell homing and targeting

Optimizing the tumor homing capacity of γδ T cells represents a major therapeutic challenge, as adoptively transferred cells frequently exhibit preferential accumulation in nontarget organs like lungs, liver, and spleen rather than tumor sites. To address this limitation, researchers have developed innovative delivery strategies, including liposome-based carriers that significantly enhance γδ T cell tumor accumulation in melanoma xenograft models [143]. Beyond homing challenges, critical technical hurdles remain in gene transfer methodology, where current approaches like retroviral transduction and EP can impair cellular viability and function. Future advancements must focus on refining gene delivery systems to preserve γδ T cell biology while improving efficiency. While CAR-γδ T cell therapy demonstrates considerable therapeutic promise, realizing its full potential will require continued technological innovation and rigorous clinical validation to establish both durable efficacy and long-term safety.

### CAR-M

CAR-M therapy has become a promising new approach to cancer immunotherapy, attracting significant research attention due to its unique therapeutic potential. While demonstrating considerable antitumor capabilities, this innovative approach faces several translational challenges that must be addressed for successful clinical implementation. Key limitations currently include constrained phagocytic activity, susceptibility to TME-mediated immunosuppression, potential off-target effects, and suboptimal *in vivo* expansion and persistence. In response to these hurdles, recent scientific efforts have developed multiple optimization strategies aimed at simultaneously enhancing both the therapeutic efficacy and safety profile of CAR-M interventions.

#### Enhancing the antitumor ability of CAR-M

One of the key roles of CAR-M cells in tumor immunotherapy is to eliminate tumor cells through phagocytosis. However, despite CAR-M’s strong phagocytic ability, its effectiveness is still limited by phagocytic efficiency and tumor cell escape mechanisms. To improve the functional potency of CAR-M cells, various optimization approaches have been developed. First, the introduction of phagocytosis-related domains (such as Megf10) can enhance the recognition and phagocytosis of tumor cells by CAR-M. These domains can increase the receptor activity of macrophages, thus enhancing their phagocytic activity [45]. Furthermore, researchers have genetically engineered CAR-M to secrete antitumor cytokines or immune stimulatory molecules (such as IL-12), which promote antigen presentation and activate other immune cells, further enhancing CAR-M’s antitumor effects [144].

Additionally, a study demonstrated that the combination of anti-CD47 and anti-HER2 antibodies holds significant promise for eliminating HER2 + breast cancer cells, mainly by enhancing macrophage-mediated antibody-dependent cellular phagocytosis (ADCP) [145]. These results indicate that CAR-M in conjunction with trastuzumab may also represent a promising therapeutic strategy in the future.

#### Overcoming immune suppression in the TME

The TME is a key factor in tumor immune evasion, especially in solid tumors, where immune suppressive factors (such as TGF-β) [146] and immune suppressive cells (such as myeloid-derived suppressor cells) [147] often inhibit the activity of immune cells and limit the efficacy of treatment. The functional failure and immune suppression of CAR-M cells in the TME is one of the major challenges in their application. To overcome this limitation, several optimization approaches have been developed. First, CAR-M cells have been engineered to express MMPs, enabling ECM degradation in the TME and improving immune cell infiltration as well as antitumor activity [47]. Moreover, studies have shown that combining immune checkpoint inhibitors, including anti-PD-1 antibodies, can overcome the immunosuppressive barriers within the TME, consequently improving CAR-M cells’ antitumor efficacy [148].

#### Improving the safety of CAR-M and reducing off-target toxicity

Translating CAR-M therapy into clinical application remains limited by considerable safety concerns, particularly concerning off-target toxicity and cytokine-mediated adverse effects. A key concern arises from the possibility that engineered macrophages may misidentify normal tissue antigens, and their systemic distribution further amplifies the risk of damaging healthy tissues. This risk is further compounded by the central role of macrophages in CRS, with IL-6 secretion being particularly problematic. Research demonstrates that CAR design significantly influences this risk profile, as evidenced by the substantially lower IL-6 levels observed in CAR-147 macrophage-treated mice compared with those receiving CAR-iMac therapies [149]. To mitigate these safety concerns, researchers have developed multi-faceted optimization approaches. First, advanced tumor antigen discovery techniques, including single-cell RNA sequencing and immune peptidomics, enable identification of highly specific tumor-associated antigens (TAAs) to minimize off-target effects [150]. Second, nonviral platforms like mRNA systems avoid viral integration risks without compromising therapeutic efficacy [114]. These strategies collectively address the dual challenges of target specificity and cytokine toxicity that currently limit CAR-M applications.

#### Improving the expansion and persistence of CAR-M *in vivo*

Improving the persistence and functional potency of CAR-M cells represents a critical challenge in macrophage-based immunotherapy, as these cells demonstrate limited *in vivo* expansion capacity compared with CAR-T cells [151]. To overcome this limitation, researchers have developed several innovative strategies. One promising approach involves utilizing stem cell-derived macrophages or genetically modified precursor cells, which exhibit superior survival and expansion properties [150]. For instance, hematopoietic stem cell (HSPC)-derived CAR-M cells from UCB demonstrate enhanced persistence and proliferative capacity *in vivo* [151]. Another strategy focuses on repeated CAR-M cell infusions to compensate for their finite lifespan and maintain sustained antitumor activity. Notably, Dong et al. achieved significant progress by reprogramming M2-like peritoneal macrophages from individuals with gastric carcinoma into M1-like CAR-M cells, which showed both prolonged survival and potent tumoricidal effects [115]. While these challenges remain substantial, the continuous advancement of CAR-M technology, coupled with emerging innovations in cell engineering, positions this therapy as a promising frontier in cancer treatment with potential applications across multiple disease types.

### Limitations and future directions

Although innovative approaches such as cytokine support, gene editing, and combination therapies have been explored to tackle the challenges of CAR-NK, CAR-γδ T, and CAR-macrophage platforms, key challenges remain. For CAR-NK cells, enhancing persistence without inducing toxicity or uncontrolled proliferation is unresolved. CAR-γδ T cells face obstacles in scalable production, subpopulation selection, and maintaining *in vivo* functional stability. CAR-macrophages must balance safety, avoidance of excessive inflammatory responses, and long-term survival for effective clinical application. Future studies should integrate systems biology approaches to optimize engineering strategies, utilize models that closely mimic human TME, and ultimately determine whether these alternative CAR platforms can achieve durable efficacy and safety in clinical settings.

## Conclusions

CAR-T cell therapy has achieved groundbreaking clinical outcomes in hematologic malignancies while facing considerable challenges in solid tumors, including off-target effects and therapeutic resistance. This review outlines the emerging potential of alternative CAR-engineered immune effectors—particularly NK cells, γδ T cells, and macrophages—as viable allogeneic platforms for next-generation immunotherapy. CAR-NK cells offer distinct advantages through their inherent MHC-independent cytotoxicity and reduced GvHD risk, though limitations in manufacturing scalability and solid tumor penetration require resolution. γδ T cells represent a unique bridge between innate and adaptive immunity, yet their clinical translation is hampered by cellular heterogeneity and persistence challenges. CAR-M demonstrates superior tumor tropism and microenvironment remodeling capacity, positioning them as promising candidates for solid tumor immunotherapy. A comparative summary of the key characteristics, advantages, challenges, and clinical progress of these three approaches is provided in Table 3,3. Emerging approaches like CAR-NKT, CAR-MAIT, and CAR-CIK cells further diversify the immunotherapy landscape. While these alternatives show great potential, comprehensive research remains crucial to optimize their safety and efficacy, collectively advancing the next generation of cancer immunotherapies beyond conventional CAR-T limitations.

**Table 3:** Summary of Key Features of CAR-NK, CAR-γδT, and CAR-M Therapies

Category	CAR-NK	CAR-γδT	CAR-M
Cell source	Peripheral blood NK cells, cord blood NK cells, NK-92 cell line, iPSC-derived NK cells	Peripheral blood γδ T cells, iPSC-derived γδ T cells	Monocyte-derived macrophages, iPSC-derived macrophages
Engineering complexity	Moderate difficulties in expansion and maintaining viability	High challenges in expansion, transduction efficiency, and heterogeneity	High requires differentiation control, phenotype stability, and delivery systems
Antitumor mechanisms	CAR-dependent cell killing, direct cytotoxicity via perforin/granzymes, ADCC through CD16, cytokine release (e.g. IFN-γ, TNF-α)	CAR-dependent cell killing, broad tumor recognition, antigen presentation, cytokine release, ADCC	Phagocytosis of tumor cells, antigen presentation, secretion of pro-inflammatory cytokines, remodeling of tumor microenvironment
Advantages	Low risk of GvHD, natural recognition of tumor cells, abundant cell sources, potential for off-the-shelf products	MHC-independent recognition, Multiple killing mechanisms, effective against diverse tumor types, potential activity in solid tumors	Ability to reshape immunosuppressive tumor microenvironment, strong innate immunity, potential synergy with other immunotherapies; Abundant infiltration in solid tumor
Challenges	Limited in vivo persistence, large-scale expansion, cryopreservation and transport issues, poor tumor infiltration	Expansion yield, transduction efficiency concerns, immunosuppressive TME has a significant impact on persistence and cytotoxic activity	Efficient delivery into tumors, maintaining function in immunosuppressive TME, potential for off-target inflammation, difficult to transduce with virus
Clinical progress	Several clinical trials with encouraging safety and efficacy data and no approved therapy	Early-stage clinical exploration, limited but growing number of trials	Mostly preclinical and early-phase clinical studies

## Open Access

This article has been published open access under our Subscribe to Open programme, made possible through the support of our subscribing institutions, learn more here: https://portlandpress.com/pages/open_access_options_and_prices#conditional

## Abbreviations

ADCC: antibody-dependent cellular cytotoxicity
ADCP: antibody-dependent cellular phagocytosis
AML: acute myeloid leukemia
BMDM: bone marrow-derived macrophages
CAR: chimeric antigen receptor
CAR-M: CAR-macrophages
CCCR: chimeric costimulatory converting receptor
CCR: chimeric co-stimulatory receptor
CCR7: chemokine receptor 7
CR: complete remissions
CRS: cytokine release syndrome
DLTs: dose-limiting toxicities
ECM: extracellular matrix
EP: electroporation
EPCR: endothelial protein C receptor
FAS: first apoptosis signal receptor
FASL: Fas ligand
GvHD: graft-versus-host disease
HSPC: hematopoietic stem cell
ICANS: immune effector cell-associated neurotoxicity syndrome
IPP: isopentenyl pyrophosphate
LNPs: lipid nanoparticles
MMP: matrix metalloproteinases
NK: natural killer
NSCAR: non-signaling CAR
ORR: objective response rate
OS: overall survival
PB: peripheral blood
PEG: polyethylene glycol
PFS: progression-free survival
SB: Sleeping Beauty
SD: stable disease
TAAs: tumor-associated antigens
TCR: T-cell receptors
TME: tumor microenvironment
TRAIL: tumor necrosis factor-related apoptosis-inducing ligand
UCB: umbilical cord blood
hPSCs: human pluripotent stem cells
iPSCs: induced pluripotent stem cells
scFv: single-chain variable fragment
